# Cardiovascular disease burden in children with obesity: A narrative review

**DOI:** 10.1016/j.ijcrp.2026.200634

**Published:** 2026-04-09

**Authors:** Sulafa K.M. Ali, Bashir H. Elnaem, Mohamed A. Abdullah, Emily M. D'Agostino

**Affiliations:** a Pediatric Cardiology, University of Sharjah, United Arab Emirates; b Pediatric Endocrinologist, Al Qassimi Women's & Children's Hospital, United Arab Emirates; c Pediatric Endocrinology, University of Khartoum, Sudan; d Department of Orthopedic Surgery, Duke Clinical Research Institute, Duke University School of Medicine, Durham, NC, United States

**Keywords:** Obesity, Children, Cardiovascular

## Abstract

Childhood obesity is increasing alarmingly and cardiovascular disease (CVD), a major complication of obesity, is documented to begin early during childhood and has detectable, preventable stages. The current guidelines for mitigating CVD prevalence do not incorporate tests for diagnosis of subclinical atherosclerosis.

A literature search under the key words “obesity, children, cardiovascular, subclinical atherosclerosis, vascular health, cardiometabolic” was conducted to cover the period from 2010 to 2025 in PubMed and the Cochrane Database of Systematic Reviews for articles published up to October 1, 2025, using combinations of terms such as “child”, “adolescent”, “obesity”, “epidemiology”, “etiology”, “complications”, “cardiovascular”, “treatment”, “atherosclerosis”, “prevention”, “endothelial function”, “pharmacotherapy”, and “BMI”. Data about the diagnosis, and management of obesity and its CVD complications were summarized in this paper.

Atherosclerosis is documented to have detectable subclinical, potentially reversible stages during childhood. The current guidelines for risk stratification do not account for the cumulative vascular burden of obesity leaving early subclinical stages undetected. While tools such as carotid intima-media thickness and carotid-femoral pulse wave velocity show clear associations with obesity and cardiometabolic risk, there is still lack of consensus on their use in routine care. More research is needed to identify tools that can be incorporated into clinical guidelines for diagnosis of subclinical CVD in children with obesity.

## Highlights

• The global prevalence of childhood obesity is increasing to alarming levels.
• Cardiovascular complications of obesity start during childhood,have a cumulative effect, and are potentially reversible.
• Pulse wave velocity is a promising tool for early detection of endothelial dysfunction; further research is required to optimize its application for children.
• The current guidelines for risk stratification fail to account for the subclinical stages and cumulative vascular burden of obesity.
• Cardiovascular health promotion using innovative tools such as mobile digital devices is critically needed in youth.

## 1 Introduction

Cardiovascular disease (CVD) remains the most common cause of death worldwide. The prevalence doubled from 271 million to over 500 million from 1990 to 2019. Death due to CVD increased by over 50%, rising from 12 million to over 18 million cases in the same period [1].

CVD in adults is dominated by atherosclerosis which is a chronic inflammatory disease resulting from genetic along with environmental factors such as hyperlipidemia, diabetes mellitus, hypertension, tobacco consumption and physical inactivity. Obesity is a classical risk factor for atherosclerosis that is implicated in pathophysiology as well as the acceleration of its complications; the most common of which are myocardial infarction and stroke [2].

Once the clinical manifestations of atherosclerosis are evident, the disease is already well established and incurable; therefore, primary prevention through lifestyle modification and early detection through screening, risk stratification and management of the disease are crucial. Obesity, as well as many of the CVD risk factors, are modifiable and potentially reversible when targeted by lifestyle and medical interventions [3].

Children with obesity are at a high risk of remaining obese as adults. It was found that 56% of children with obesity and 80% of children with severe obesity become adults with obesity which imposes a cumulative metabolic and cardiovascular risk on these patients. [4]. Childhood obesity increases the risk of adult CVD due to the cumulative effect over time**.**

It was shown that children in the highest quartile of body mass index (BMI) had more than double the rate of premature death, including CVD, than those with the lowest BMI quartile. [5]. The International Childhood Cardiovascular Cohort Consortium studied 5 risk factors for atherosclerosis in a cohort of 38589 participants with a follow-up of 35 years. This study demonstrated a strong association of childhood risk factors with major CVD events [6].

Although the cardiovascular complications of obesity are known to begin during childhood, the early detection of subclinical stages has been underreported and undertreated. Several factors may contribute to this, including the difficulty of documenting atherosclerosis at its earliest stage and the lack of consensus regarding the best method of screening.

Moreover, since the incidence of clinical CVD is much higher in older age, most young patients, particularly children, would not fulfill the classic criteria for initiating screening for atherosclerotic diseases. This creates a large population of high-risk young adults, and potentially children, untreated [7].

There is a global move towards identifying discrete subsets of atherosclerosis, aiming to prevent clinical events. A global initiative defined a novel precision medicine-driven screening program, including imaging and biomarkers for early detection. [8]. Such programs are potentially applicable to children with risk factors.

This review aims to summarize current evidence for the early cardiovascular effects of childhood obesity, evaluate available tools for detecting subclinical atherosclerosis, and identify research and practice gaps.

## 2 Search strategy and selection criteria

1. References for this Review were identified through searches of PubMed and the Cochrane Database of Systematic Reviews for articles published up to October 1, 2025, using combinations of terms such as “child”, “adolescent”, “obesity”, “epidemiology”, “etiology”, “complications”, “cardiovascular”, “treatment”, “atherosclerosis”, “prevention”, “endothelial function”, “pharmacotherapy”, and “BMI”. Studies were selected based on critical reviews of their methodologies, with preference for recent (last 5-10 years) meta-analysis and randomized controlled trials. Articles published in English were included. We also reviewed reference lists of published manuscripts, clinical guidelines, and other relevant reviews and meta-analyses.

## 3 Definition and epidemiology of obesity and CVD in children

Obesity is defined as a pathological condition with abnormal or excessive fat due to complex interactions between genetic, environmental, and lifestyle factors.

BMI is the most commonly used simple measure employed to classify overweight and obesity. BMI categories for children and teens are based on sex-specific BMI-for-age percentiles.

However, there are limitations to using BMI as it does not account for muscle mass and bone density; therefore, a better definition of obesity is measuring waist circumference, waist to hip or waist to height ratio in addition to BMI [9].

Overweight is defined as a BMI between the 85th and 95th percentiles; obesity is defined as a BMI at or above the 95th percentile; and severe obesity is defined as a BMI more than 120% of the 95th percentile or 35 kg/m2. Severe obesity is further classified as class 2 and 3 defined as 120% to less than 140% and 140% or more of the 95th percentile, respectively. [10]. In a recent cross-sectional study of US children and adolescents, two more classes were described for severe obesity, referred to as class 4 (BMI 160% to <180% of 95th percentile)and class5 (BMI = /> 180% of 95th percentile) [11].

The Commission on the definition and diagnostic criteria of clinical obesity recently released a new classification distinguishing “clinical” and “preclinical obesity”. Clinical obesity is defined as a chronic systemic disease caused by excessive adipose tissue, while preclinical obesity is defined as excessive adiposity without organ dysfunction [12].

The prevalence and severity of obesity are alarmingly increasing globally and across all age groups, reaching a level of an epidemic proportions with no signs of slowing. The combined prevalence of overweight and obesity in children and adolescents doubled between 1990 and 2021, and that of obesity alone tripled. In 2021, 93 million individuals aged 5-14 years and 80 million aged 15-24 years were affected by obesity. Obesity and overweight, therefore, are considered the most common chronic diseases in children. The disability-adjusted life years rates attributable to obesity are projected to increase from 1946 to 2099/100 1000 people. Moreover, death rates are projected to rise from 62 to 64 per 100 000 people in 2030 [13].

Analysis of data from 68 million individuals, including children, for over 25 years, found that the rate of increase in childhood obesity is greater than that in adults. A high BMI accounted for 4 million deaths; 2 thirds of deaths were due to CVD [14].

## 4 Pathophysiology of CVD in obesity

The hemodynamic alterations related to obesity that are involved in the mechanisms of CVD and the early detectable manifestations in children are shown in Table 1. Although most data are derived from adults, the same pathogenic mechanisms are expected to evolve in children with obesity, ultimately leading to CVD. The manifestations of CVD are largely subclinical, becoming apparent only in advanced stages. Systemic hypertension is the earliest cardiovascular clinical sign that was reported in up to 21% of children with obesity. Other manifestations are subclinical [15,16].

**Table 1:** Mechanisms involved in the pathogenesis of CVD.

Hemodynamic Alteration	Effect	Clinical Manifestations (Mostly in adults)	Early Manifestations in Children
Increased blood volume.^15^	Increased blood pressure Left ventricle dilatation and systolic dysfunction	Left ventricle remodeling, dilated heart with potential cardiomyopathy,	Endothelial dysfunction Hypertension Left ventricle hypertrophy Left ventricle diastolic dysfunction
Increased cardiac output and blood pressure^16^	Vascular complications in different organs Diastolic ventricle dysfunction	Stroke Heart failure	Endothelial dysfunction Hypertension Left ventricle hypertrophy Left ventricle diastolic dysfunction
Arterial Stiffness^16^	Atherosclerosis	-Coronary artery disease -Vascular complications in different organs	Endothelial dysfunction
Increased peripheral resistance	-Increase left ventricle after load and myocardial stress	Heart failure	Endothelial dysfunction Hypertension Left ventricle hypertrophy Left ventricle diastolic dysfunction
Pulmonary Hypertension	Usually secondary to obstructive sleep apnea	Aggravation of heart failure	Echocardiographic findings of pulmonary hypertension

## 5 The burden of CVD in children with obesity

The burden of CVD is influenced by the following factors:

### 5.1 Cumulative, age-related effect of risk factors for CVD in children with obesity

The individual traditional risk factors for CVD such as diabetes, hypertension and smoking had been well studied both in children and in adults. The danger of cardiovascular complications is their silent, cumulative nature. In a recent cohort of 4832 young adults followed up for 35 years, the cumulative Life's Essential 8 score from age 18 to 45, independent of the score at 45 years and the rate of improvement from 18 to 45 years, was shown to have a significant association with lower CVD hazards. This indicates that improvement of cardiovascular health during young adulthood is independently associated with lower risk for CVD in midlife [17].

Another study for low-density lipoprotein level in young adults showed that cumulative lipoprotein levels during young adulthood are a risk for coronary artery disease in adults [18].

The Cardiovascular Risk in Young Finns study examined carotid plaque in 2643 adults who had a history of dyslipidemia in childhood. Carotid plaque area was found to correlate with childhood total low -density lipoprotein cholesterol levels. The study concluded that childhood dyslipidemia, even when resolved by adulthood, is a risk factor for carotid plaque, highlighting the need for early interventions during childhood [19].

### 5.2 Clustering of multiple risk factors

Children's cardiovascular health has been defined by the American Heart Association by eight parameters: diet, physical activity and screen time, sleep, tobacco exposure, BMI, blood pressure, cholesterol, and glucose measurements [20]. Obesity is closely interacting with most of these parameters; therefore, it has a central role in children's cardiovascular health and leads to clustering of CVD risk factors in the same patient. A classic example is the metabolic syndrome. (MS) which is a disorder characterized by abdominal obesity, high blood pressure, hyperlipidemia, and high fasting blood glucose. While obesity independently poses a risk for CVD, it is also a precursor of MS, which further accelerates this risk. There is no consensus on the definition of MS in pediatric age. The most widely accepted definition of the International Diabetes Foundation. Central obesity (waist circumference ≥90th percentile for age and sex)is a prerequisite; then 2 out of 4 criteria are needed for diagnosis of MS in children over 10 years old, including [21]:

1. Fasting triglyceride blood level ≥150 mg/dL (1.7 mmol/l)
2. Fasting high density lipoprotein cholesterol blood level ≤40 mg/dL (1.03 mmol/l)
3. Systolic or diastolic blood pressure ≥130 mmHg or diastolic blood pressure≥ 85 mmHg
4. Fasting blood glucose ≥100 mg/dL (5.6 mmol/l) or the presence of type 2 diabetes.

**Fig. 1:**
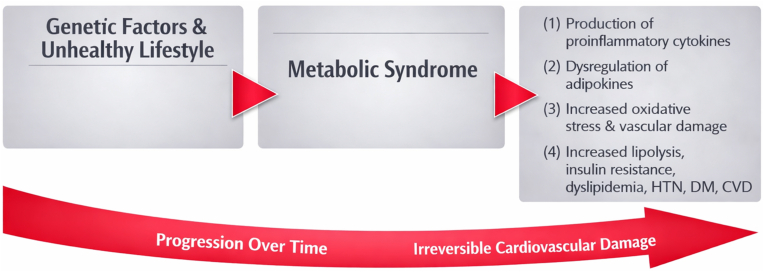
Causes and Consequences of MS.

The prevalence of MS varies between countries depending on the definition used. The pathophysiology of MS involves genetic predisposition coupled with an unhealthy lifestyle leading to obesity [22]. The causes and consequences of MS are shown in Fig. 1. As a result of the MS, the risk of CVD in obese individuals becomes accelerated due to the synergistic and overlapping nature of the pathogenesis of MS with that of CVD.

### 5.3 Silent stages of CVD in fetuses, infants, and children exposed to high-risk factors

**Fig. 2:**
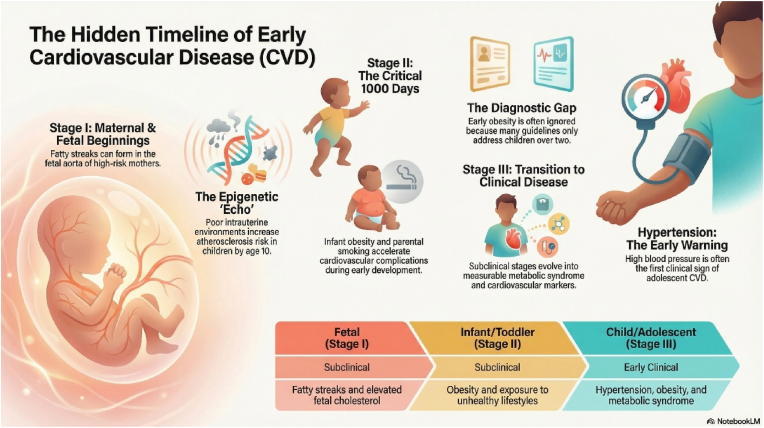
Stages and features of CVD in children exposed to risk factors (including obesity) (Produced using NotebookLM).

The myths that CVD is associated with old age have been disputed by studies that showed lipid streaks during fetal life when the pregnant lady has risk factors for atherosclerosis. Fig. 2 shows the stages of the disease.

#### 5.3.1 Stage 1: maternal and fetal

The earliest evidence of obesity-related CVD in children was found in studies of fetuses of hypercholesterolemic mothers. Fetal cholesterol level was elevated, and there was fatty streak formation in the aorta with established atherosclerosis in 58% of the lesions [23]. These babies exhibited progression of atherosclerosis during childhood even in the absence of hypercholesterolemia, [24].In addition, epigenetic adaptation due to an unfavorable intrauterine environment leads to increased risk of atherosclerosis in children born to mothers with CVD risk factors. This was shown in a large multinational study of mothers who had high CVD risks; their offspring had poor cardiovascular health parameters at the age of 10-14 years [25].

#### 5.3.2 Stage 2: infants and toddlers

Obesity in the first 1000 days of life (neonates and infants) is a risk factor for the disease later in childhood and in young adults. At this stage, external factors such as an unhealthy diet and parental smoking will be imposed on the existing genetic and epigenetic factors, all leading to the progression of CVD complications. Obesity in this period starts to manifest clinically; however, this is often ignored by parents and medical staff. Most protocols address the management of obesity in patients more than 2 years of age; therefore, the prevention of obesity at this stage needs to be emphasized and included in guidelines [26].

#### 5.3.3 Stage 3: children and adolescents

Children and adolescents with obesity can have a subclinical stage of CVD. This stage can be identified by blood and imaging investigations (see below). Progression of this phase results in clinical signs of CVD, the earliest of which is HTN, which can be present with other features of MS.

## 6 Diagnosis of subclinical atherosclerosis

According to the American Heart Association (AHA)classification, children with high CVD risk include familial hypercholesterolemia, Kawasaki disease with persistent coronary aneurysms and childhood cancer. Children with severe obesity were classified as “moderate CVD risk “while children with obesity and insulin resistance are classified as “at risk” [27]. However, this risk stratification does not take into consideration the age-related cumulative effects and clustering of risk factors in children with obesity. Moreover, this risk classification fails to account for the silent/subclinical stages (1 and 2) of atherosclerosis. For all the risk categories, AHA recommends screening for the conventional risk factors (blood glucose, lipid profile, blood pressure, and physical activity level). There is currently no consensus regarding screening for subclinical atherosclerosis.

Endothelial dysfunction (ED) is a process that precedes atherosclerotic CVD and is defined as a series of maladaptive vascular changes that lead to loss of the endothelium's capacity to promote vasodilation, fibrinolysis, and anti-aggregation. This leads to oxidative stress, vascular inflammation, and progression of atherosclerosis [28].ED is an early sign of CVD, it's measurable and reversible, therefore it is potentially an ideal target for CVD prevention in children with risk factors. Carotid intima-media thickness (cIMT) measured by ultrasound and arterial stiffness, measured by the carotid-femoral pulse wave velocity (cf PWV), were identified as important markers of subclinical atherosclerosis in young adults. [[29], [30], [31]]. Although there is a strong correlation between BMI and a high cIMT in children together with the available age and sex matched normative values [32], there is still ongoing debate regarding its routine clinical utility. Limitations to clinical application include wide variation between ethnic groups and intra-observer variability in ultrasound techniques, leading to limited reproducibility [33].

cfPWV, which measures the speed of propagation of the arterial pulse from carotid to femoral artery, has a strong association with CVD risk in adults and is considered the gold standard for assessment of arterial stiffness. In a recent study including 48 257 adults aged 20 and older, elevated PWV was found to be independently associated with increased cardiovascular mortality. [34]. This finding strongly underscores the value of this test for risk stratification.

In children, cfPWV is gaining attraction because it is non-invasive and has been proven to correlate with CVD risk in children with obesity, diabetes, and dyslipidemia [35].

A systematic review of the association of PWV with metabolic risk factors in children revealed a positive correlation between cfPWV and impaired glucose metabolism and metabolic syndrome; however, the correlation with obesity was not consistent [36].

Limitations of PWV use in pediatric age include the lack of reference values for a large multi-ethnic population. Moreover, there are ethical concerns in correlating PWV with cardiac catheterization measurements due to the invasive nature of this investigation.

Despite the extensive research that documented the role of cIMT and cf PWV in detection of subclinical atherosclerosis, their clinical implementation is not yet evidence-supported. More research is needed to identify age, sex, and ethnic reference values and refine the techniques’ reproducibility. Long-term longitudinal studies are needed to correlate the current abnormal childhood endothelial dysfunction with CVD mortality in adulthood [37].

Echocardiography can assess left ventricle (LV) hypertrophy, systolic and diastolic function; however, abnormal findings are usually seen in advanced CVD. The best measure to define left ventricular hypertrophy is debatable. M-mode measurement of LV hypertrophy is defined by a value equal to or more than 3 standard deviations above the mean for surface area.LV mass correlates well with BMI and biochemical parameters and tends to improve with weight control. LV [38,39].

It is recommended to do an echocardiography examination in children with obesity in the presence of comorbidities, including hypertension, renal failure, dyslipidemia, or type 2 diabetes [33].

## 7 Management of obesity and its impact on CVD

### 7.1 Primordial and primary prevention: multisectoral and community-based approaches

A community -based team that includes public health organizations, schools, parks and recreation, and neighborhood community-based organizations is highly needed for effective control of obesity. Simple measures such as providing playgrounds might have an important impact on reducing obesity and its CVD [40]. Community-level programs are integral to reducing CVD risk factors among youth and establishing healthy habits across the lifespan. Such programs were found to be effective in improving public awareness about CVD [41]. Lifestyle interventions such as improving physical activity and nutrition in children with obesity were found to be effective in reducing obesity-related CVD. [42]. Impactful advancements in cardiovascular promotion have been achieved by using digital applications and wearable devices. These were shown to improve heart rate variability in high-risk adults [43]. Using a mobile application for monitoring of physical activity, body weight, and dietary habits was shown to improve atherosclerosis burden in adults. [44]. Mobile technology can be particularly attractive in youth. The awareness material can be adjusted to be age-specific, and it can simultaneously positively impact both adolescents and their families.

#### 7.1.1 Tertiary prevention of CVD

A comprehensive evaluation of the child with obesity by a pediatrician with special training in endocrinology/obesity is mandatory. The severity/class of obesity and risk stratification will dictate the screening pathway for CVD. Accordingly, a plan of management is designed with consideration of the child's medical, social, and psychological circumstances. A multidisciplinary hospital team, including physicians, social workers, dieticians, and psychologists, is imperative.

#### 7.1.2 Nonpharmacological management

Recently, the Canadian Clinical Practice Guidelines recommended using multicomponent tools together with dietary and exercise interventions to manage children with obesity. These include psychological/behavioral interventions as well as technology-derived tools such as mobile phone interactive applications [45]. As obesity can be a source of oxidative stress, many natural anti-inflammatories and antioxidants have been tried, such as prebiotics and vitamins. Selenium plays a crucial role in neutralizing free radicals, and it has been identified, together with other antioxidants such as vitamin C and E as an indicator of the body's compromised ability to combat oxidative stress in obesity [46]. Emerging studies suggest a role for antioxidants in improving endothelial function and HTN in children with obesity; however, more studies are needed to investigate the safety and long-term effects of this therapy [47].

#### 7.1.3 Pharmacological treatment

Statins had been used effectively to treat hyperlipidemia and proved to reduce IMT progression and atherosclerosis burden in children [48]. Similarly, metformin is shown to improve IMT and inflammatory markers [49]. Sodium-glucose co-transporter-2 inhibitor was shown to mitigate the metabolic derangements and their impact on endothelial function in adults. This drug had been used for children and young adults with type II DM and proved to be safe and well tolerated; therefore, it could have a potential role in prevention of early CVD [50]50. More studies are needed to investigate such a role.

Currently, 4 anti-obesity drugs are approved for children >12 years of age: orlistat, liraglutide, semaglutide and phentermine-topiramate. The use of medications needs to be coupled with lifestyle modification, particularly diet and physical exercise. Semaglutide and phentermine/topiramate are effective in reducing BMI; however, there is still a knowledge gap regarding the long-term effects on CVD [51]. Barriers to studying the long-term effects in the pediatric age include the long time needed for follow-up and the prevalence of multiple confounding factors during this long time.

#### 7.1.4 Surgical interventions

Surgery for obesity include Roux-en-Y gastric bypass and vertical sleeve gastrectomy, indicated for BMI≥120% of the 95th percentile. It was found to be effective in reducing BMI by about 30%, however, the complication rate may reach 15%. While cardiovascular outcomes appear favorable within the first two years, further studies are required to determine long-term effects [52].

#### 7.1.5 Conclusion and future perspectives

• CVD linked to pediatric obesity has detectable, potentially reversible, subclinical stages; yet current guidelines do not include tools for its early detection. This failure to screen leaves many children at a high risk of progression into adolescence and adulthood with unrecognized vascular disease.
• . Improving early detection is expected to **reduce the clinical burden** of CVD and significantly **alter its lifelong trajectory** in this population.
• While there are available tools for diagnosis of subclinical atherosclerosis,their use in routine practice is limited by technical variability, lack of pediatric normative data, and insufficient validation.
• Digital tools and mobile devices are expected to have a significant role in cardiovascular health promotion in youth.

**More research is needed to**:

• Establish standardized reproducible protocols and large, multi-ethnic reference datasets for cIMT and cf PWV.
• Correlate childhood subclinical CVD with adult outcomes through longitudinal cohort studies
• Innovate smart devices, using artificial intelligence, that can predict CVD risk and detect early atherosclerosis.
• Evaluate the impact of anti-obesity therapies on vascular function in children.

## CRediT authorship contribution statement

**Sulafa K.M. Ali:** Writing – review & editing, Writing – original draft, Project administration, Methodology, Data curation, Conceptualization. **Bashir H. Elnaem:** Writing – review & editing, Writing – original draft, Data curation. **Mohamed A. Abdullah:** Writing – review & editing. **Emily M. D'Agostino:** Writing – review & editing.

## Declaration of generative AI and AI-assisted technologies in the manuscript preparation process

During the preparation of this work, the authors used [NotebookLM] to generate the image (Fig. 2). After using this tool/service, the authors reviewed and edited the content as needed and took full responsibility for the content of the published article.

The following abbreviations are used in this manuscript.

## Abbreviations

AHA: American Heart Association
BMI: Body Mass Index
cfPWV: carotid-femoral pulse wave velocity
CVD: Cardiovascular Disease
ED: Endothelial dysfunction
HTN: Hypertension
IMT: Intima-Media Thickness
DM: Diabetes Mellitus
MS: Metabolic Syndrome
